# Basosquamous Carcinoma: Comprehensive Clinical and Histopathological Aspects, Novel Imaging Tools, and Therapeutic Approaches

**DOI:** 10.3390/cells12232737

**Published:** 2023-11-30

**Authors:** Giulia Murgia, Nerina Denaro, Francesca Boggio, Gianluca Nazzaro, Valentina Benzecry, Paolo Bortoluzzi, Emanuela Passoni, Ornella Garrone, Angelo Marzano

**Affiliations:** 1 Dermatology Unit, Fondazione IRCCS Ca’ Granda Ospedale Maggiore Policlinico, 20122 Milan, Italy; gianluca.nazzaro@policlinico.mi.it (G.N.); valentina.benzecry@policlinico.mi.it (V.B.); paolo.bortoluzzi@policlinico.mi.it (P.B.); emanuela.passoni@policlinico.mi.it (E.P.); angelo.marzano@unimi.it (A.M.); 2 Oncology Unit, Fondazione IRCCS Ca’ Granda Ospedale Maggiore Policlinico, 20122 Milan, Italy; nerina.denaro@policlinico.mi.it (N.D.);; 3 Department of Pathology, Fondazione IRCCS Ca’ Granda Ospedale Maggiore Policlinico, 20122 Milan, Italy; francesca.boggio@policlinico.mi.it; 4 Department of Pathophysiology and Transplantation, Università degli Studi di Milano, 20133 Milan, Italy

**Keywords:** basosquamous carcinoma, metatypical basal cell carcinoma, diagnosis, treatment, biologic behavior, dermoscopy, LC-OCT, histopathology, Mohs’ micrographic surgery, genetics, vismodegib, sonidegib, cemiplimab, nivolumab, pembrolizumab

## Abstract

Basosquamous carcinoma (BSC), an uncommon and aggressive nonmelanoma skin cancer exhibiting characteristics ranging from basal cell carcinoma (BCC) to squamous cell carcinoma (SCC), is a subject of controversy in terms of its classification, pathogenesis, histologic morphology, biologic behavior, prognosis, and management. This narrative review is based on an electronic search of English-language articles in PubMed that included the terms “basosquamous carcinoma” and/or “metatypical carcinoma of the skin” in their titles. The review aims to succinctly present and assess current data on the epidemiology, clinical presentation, dermoscopic, LC-OCT, and histopathologic characteristics, as well as the genetics and management of BSC, providing insight into this intriguing entity. As a conclusion, dermoscopy, deep incisional biopsies, and immunohistologic techniques should be applied in clinically suspicious lesions to achieve an early diagnosis and better prognosis of this tumor. Surgical treatments, including wide excision and Mohs’ micrographic surgery, remain the treatment of choice. Finally, Hedgehog pathway inhibitors and checkpoint inhibitors, must be thoroughly investigated with large controlled trials, since they may offer an alternative solution to irresectable or difficult-to-treat locally advanced cases of basosquamous carcinoma.

## 1. Introduction

Basosquamous carcinoma (BSC), also known as metatypical basal cell carcinoma, represents a subtype of basal cell carcinoma (BCC) characterized by a combination of clinical, dermoscopic, and histologic features from both basal cell carcinoma (BCC) and squamous cell carcinoma (SCC), along with the presence of a transition zone. It is believed that BSC initially manifests as a BCC with genetic and epigenetic alterations leading to squamous differentiation through basal to squamous transition (BST). Notably, due to its local invasiveness, high recurrence rate, and metastatic potential, BSC stands out as one of the most aggressive subtypes of BCC. In contrast to other nonmelanoma skin cancers (NMSC), controversies have emerged over the years regarding the classification and therapeutic management of BSC. The World Health Organization (WHO) classifies BSC as basal cell carcinomas exhibiting both carcinomatous squamous and basaloid zones in varying proportions. However, some of the literature challenges this classification, referring to it as mixed BCC-SCC, metatypical BCC, or even a collision carcinoma. Notably, unlike other NMSC, the National Comprehensive Cancer Network (NCCN) does not provide specific guidelines for the management and treatment of BSC. This article aims to summarize and evaluate the most recent data from the English literature concerning the epidemiology, clinical presentation, confocal, dermoscopic, histopathologic characteristics, genetics, and management of BSC, contributing to a better understanding of this enigmatic neoplasm.

## 2. Materials and Methods

The terms “basosquamous carcinoma” and/or “metatypical BCC”, along with subheadings such as classification, incidence, epidemiology, diagnostics, histology, dermoscopy, genetics, biologic behavior, and treatment, were systematically searched in English-language papers published from 1910 to 2023, utilizing the PubMed database. Inclusion criteria comprised (1) case series or case reports specifically addressing basosquamous carcinoma and (2) review studies, meta-analyses, and systematic reviews focused on basosquamous carcinoma. Exclusion criteria encompassed (1) papers written in languages other than English and (2) articles discussing basosquamous carcinoma in organs other than the skin (e.g., larynx, nasopharynx, lungs, anus, etc.). In addition, chosen pieces from the included publications were used to enhance the discussion of our review.

## 3. Results

### 3.1. Definition

Beadles published the first description of BSC in 1894, classifying it as a particular kind of rodent ulcer [1,2]. He described a lesion with characteristics of both BCC and SCC that were difficult to distinguish [1,2]. Again, in 1910, McCormack wrote about carcinomas with intermixed basaloid and squamous characteristics in a wider series of rodent ulcers [2,3]. Montgomery first introduced the term “basal squamous cell carcinoma” (BSC) to refer to 17 out of 119 carcinomas in 1928, which he believed to be in a transitional stage between basal and squamous cell carcinomas [4,5].

The origin and definition of this neoplasm perplexed pathologists for many years [6,7]. Some hypothesized that these lesions resulted from collisions between distinct primary BCC and SCCs, others suggested a BCC variant producing keratin, while some considered them as different carcinomas with characteristics of both BCC and SCC [2,8,9]. Meanwhile, BSC was alternatively labeled as “metatypical carcinomas” in certain publications [5,10,11]. A more recent and widely accepted theory posits that BSC originates from a BCC undergoing squamous differentiation due to genetic changes [7,11,12]. The World Health Organization’s (WHO) latest definition of BSC in the “WHO Classification of Skin Carcinomas” aligns with this theory, stating that “Basosquamous carcinoma is a term used to describe basal cell carcinomas that are associated with squamous differentiation” [13]. However, despite this characterization, several studies have documented a more aggressive behavior [5,7,14]. According to the National Comprehensive Cancer Network (NCCN) BCC Guidelines Version 2.2024, BSCs exhibit a metastatic capability more similar to SCC than BCC [5,7,14]. Hence, clinicopathologic correlation is recommended in these cases, and patients may necessitate a distinct protocol of treatment and followup compared to BCC patients. Due to the distinct aggressive biological behavior and clinical course that sets BSC apart from other forms of BCC, some authors caution against the use of the term “metatypical basal cell carcinoma”, considering it potentially misleading [5].

### 3.2. Epidemiology

BCCs and SCCs are the most common NMSCs with a rising incidence in the general population, while BSC is considered a relatively rare entity [7]. To date, few studies have directly evaluated the epidemiology of BSC, and most of these studies were small in size due to the relatively recent histological definition of this clinical entity [5,6,15]. As reported in a previous review, the incidence of BSC varies between 1.7 and 2.7% [5]. Specifically, Shuller et al. reported an incidence of 1.2%, Martin et al. reported 1.4%, and Bowman et al. documented a 2.7% incidence in a retrospective review of cases treated with Mohs’ micrographic surgery [6,16,17]. In a more recent retrospective analysis, Ciazynska et al. diagnosed 180 cases of BSC over a 20-year period (1999–2019), corresponding to 2.1% of all NMSCs [7]. However, Gualdi et al., in a prospective study covering the years 2012–2015 and including 6042 NMSCs, reported a higher rate of 4.8% for BSCs, a percentage significantly elevated compared to previous findings [15]. Large-scale studies that enable the radical excision of the lesion with proper histological and immunohistochemical evaluation are required for a correct epidemiological analysis of BSC because the clinical diagnosis of this neoplasm is very challenging, and histological confirmation is finally required.

### 3.3. Clinical and Demographic Characteristics

The clinical appearance of BSC is notably ambiguous, with no discernible differences from a typical BCC [5,7]. In fact, a persistent nodule that eventually transforms into an ulcer represents the most characteristic clinical presentation for BSC. The most common anatomical locations are the sun-exposed areas of the head and neck, constituting 82–97% of cases, particularly affecting the perinasal region and ears [2,5,6,7,17,18,19]. While BSCs have been identified on the trunk and extremities, these occurrences are comparatively less frequent [2,5,6,7,17,18,19]. Notably, Fitzpatrick skin types I–II and high ultraviolet radiation (UVR) exposure are significant risk factors for BSC development [11]. The elderly population is more susceptible to developing this neoplasm, with individuals over 70 years old accounting for 34.4% of all BSC cases with a marked male predominance [6,12,15].

### 3.4. Diagnosis of Basosquamous Carcinoma

Recognition of this cutaneous tumor remains challenging, and clinical diagnosis is particularly demanding due to its rarity and nonspecific presentation, often characterized by the presence of a rapidly growing ‘rust-red’ ulceration [5]. Seborrheic keratosis (SK), hyperkeratotic actinic keratosis (AK), Bowen’s disease (BD), BCC, invasive SCC, and amelanotic/hypomelanotic melanoma (AHM) are a few examples of conditions that must be included in the differential diagnosis [6,17,20,21]. Histopathological analysis is considered the gold standard for diagnosing Basosquamous Carcinoma (BSC). However, occasional misclassifications of BSC as Basal Cell Carcinoma (BCC) or Squamous Cell Carcinoma (SCC) have been reported, frequently due to nonrepresentative biopsies of deeply located tumors. In this context, noninvasive skin imaging plays a crucial role in both improving BSC recognition and optimizing subsequent followup strategies [22,23,24,25,26,27,28].

#### 3.4.1. Dermoscopy of BSC

BSC appears to exhibit dermoscopic features that overlap with both Basal Cell Carcinoma (BCC) and invasive Squamous Cell Carcinoma (SCC). Detection of at least one dermoscopic criterion from both BCC and SCC categories should raise suspicion. According to Giacomel et al. [22], the most common dermoscopic characteristics of BSC include unfocused arborizing vessels (73%), typically located at the periphery of the tumors, as well as keratin masses (73%) and whitish structureless areas (73%). Other frequently observed dermoscopic features encompass superficial scales (68%); ulceration or blood crusts (68%); white structures like clods, circles, and lines (64%); and blue-grey blotches (59%). Only 27% of BSC cases presented pigmentation features resembling those of BCC, such as multiple brown dots and leaf-like areas. Focused arborizing vessels were detected in only 14% of BSC cases, and in each instance, they were part of a polymorphous vascular pattern [22]. Half of the tumors exhibited a polymorphous vascular pattern, comprising various combinations of dotted, linear irregular, and arborizing vessels. However, only one case displayed a polymorphous vascular pattern lacking arborizing vessels, featuring dotted and linear irregular vessels [22]. The study of Acay et al. [23] demonstrated that BSC is dermoscopically characterized by BCC-related polymorphous or monomorphous vasculature, combined with signs of keratinization [26]. Specifically, a serpentine of branched vessels was the most common vascular finding, while keratin masses, ulceration, and white structureless areas were the most common nonvascular features [23]. 

In a recent study, Camela et al. [24] revealed that arborizing telangiectasias (77.8%), shiny white structures (66.7%), and ulceration (62.9%) are the most frequently observed dermoscopic structures in BSC. Additionally, follicular criteria and milky red structureless regions were common dermoscopic clues, each noted in 40.7% of cases. The study also found that the percentage of BSC cases with keratin masses (29.6%) was higher than in other aggressive subtypes of Basal Cell Carcinoma (BCC) such as morpheaform, micronodular, infiltrative, and metatypical BCC [24].

#### 3.4.2. Line-Field Confocal Optical Coherence Tomography (LC-OCT) of BSC

Line-field confocal-OCT is a new promising technique that may support the noninvasive recognition of BSC through the simultaneous detection of at least one BCC-associated feature and at least one SCC-associated feature. The use of LC-OCT might be helpful not only in the diagnostic setting but also in the followup surveillance for an early identification of recurrences. However, further larger studies are needed to prove this hypothesis [26,27,28].

Oliveria et al. [26] identified LC-OCT criteria for BSC that included both BCC-associated and SCC-associated characteristics. Dermal lobules featuring the characteristic millefeuille pattern, dilated vessels, bright cells within the epidermis, bright cells within lobules, stromal stretching, and stromal brightness represented BCC-associated features [26,27]. Acanthosis, hyperkeratosis, disarranged epidermal architecture, broad strands, elastosis and glomerular vessels encompassed SCC-associated criteria. Overlapping criteria included the interruption of the dermal–epidermal junction and ulceration [28]. 

#### 3.4.3. Histopathologic Features of BSC

Deep biopsy with histologic examination remains the gold standard diagnostic method for BSC. Histologically, BSC is defined as a neoplasm containing three different areas: a BCC area, a SCC area, and a transition zone between them. However, there is ongoing disagreement among dermatopathologists regarding the arrangement of these zones within the lesions [2,5,11]. Most authors consider the transition zone as tissue depicting a transitional stage of differentiation between BCC and SCC cells, rather than simply an area with atypical BCC cells [2,5,29]. In BSC, the BCC area consists of basaloid cells with small cytoplasm and large, uniform, and pale nuclei. Conversely, the identification of the SCC area requires accumulations of polygonal squamous cells with abundant eosinophilic cytoplasm, larger open nuclei with prominent nucleoli, and frequent mitosis [2,17,29,30]. These aggregates of squamous cells are either located inside the basaloid islands or, as some authors describe, adjacent to them [2,5,23,29,31,32]. The main entities to consider in the histologic differential diagnosis of BSC are a collision tumor and a keratinizing BCC. In the case of a collision tumor, the BCC and SCC areas should represent two completely separated lesions juxtaposed with no transition zone. In the case of a keratinizing BCC, there is abrupt keratinization in the center of a nodular BCC lesion without the intervening areas of recognizable squamous cells [1,2,33]. Unfortunately, incorrect histopathologic diagnosis is common, especially when only a superficial biopsy is obtained. A deep punch or incisional biopsy is often required for a correct diagnosis, as the lesion may exhibit compromised features of BCC superficially, with BSC features becoming evident only in deeper areas of the tumor [1,2].

#### 3.4.4. Immunohistologic Features of BSC

While there is no specific immunohistochemical marker exclusively for BSCs, Ber-EP4, which is typically strongly positive in Basal Cell Carcinomas (BCCs), and epithelial membrane antigen (EMA), which stains positively in Squamous Cell Carcinomas (SCCs), have been found to be helpful in the diagnostic process [2,5,18]. Additionally, the transition zone typically exhibits a gradual decline in Ber-EP4 staining, serving as an indication of the transition from the basaloid zone to the area with squamous differentiation [2,5,34]. It is well known, however, that poorly differentiated SCC may assume a basaloid phenotype (BSCC), complicating the histological distinction between BSC and basaloid SCC (BSCC). Webb et al. [35] demonstrated that BSCs present a diffuse staining for Ber-EP4 and MOC-31, while BSCCs are only sporadically reactive for both markers. Positive stain for UEA-1 was observed in almost all BSCCs but only in few cases of BSC showing limited and focal positivity. These data suggest that MOC-31 is a useful marker in the specified differential diagnosis, especially when used together with UEA-1 [35].

According to Haensel et al. [36], surface marker Lymphocyte Antigen 6 Family Member D (LY6D) marks and tracks basosquamous populations. They show that LY6D+ tumor cells lie on a differentiation spectrum between BCC and SCCs and labels the transition area of BSC.

Regarding the immunohistochemical profile among different subtypes of BCCs, Cojocaru et al. illustrated that BSCCs exhibited higher CD31, CD34, and alpha-smooth muscle actin (α-SMA) stromal expression, suggesting a link between the tumoral microenvironment and the invasiveness (and consequently aggressiveness) of this entity [37]. 

### 3.5. Genetics and Pathogenesis

While the exact gene mutations causing BSC are still debated, the genetic background of BCC and SCC has been extensively examined. BCC is characterized by the excessive activation of the sonic hedgehog (HH) pathway, either through the inhibition of the transmembrane protein PTCH or the activation of SMO [38,39,40]. Various genetic drivers of BCC include PTEN, MYCN, PPP6C, GRIN2A, GLI1, CSMD3, DCC, PREX2, and APC [38,41,42]. SCC is distinguished by a diverse range of gene mutations, including alterations in HRAS and disruptions of genes such as TGFBR1, TGFBR2, NOTCH1, NOTCH2, CASP8, CDKN2A, NOTCH3, KRAS, NRAS, PDK1, BAP1, AJUBA, KMT2D, MYH9, TRAF3, NSD1, CDH1, and TP63 [38,43,44,45,46]. According to Chang et al. [38], a significant number of BSCs exhibited underlying PTCH1 and SMO mutations. Additionally, these BSCs showed mutations commonly associated with BCC, including MYCN, PPP6C, GRIN2A, CSMD3, DCC, PREX2, APC, PTEN, and PIK3CA [38]. These findings lend support to the hypothesis that the sonic hedgehog (HH) signaling pathway serves as the primary driver mutation in BSC. It is suggested that BSC likely originates as a BCC and undergoes partial differentiation into SCC through the accumulation of ARID1A mutations and activation of the RAS/MAPK pathway [38,47]. 

**Figure 1:**
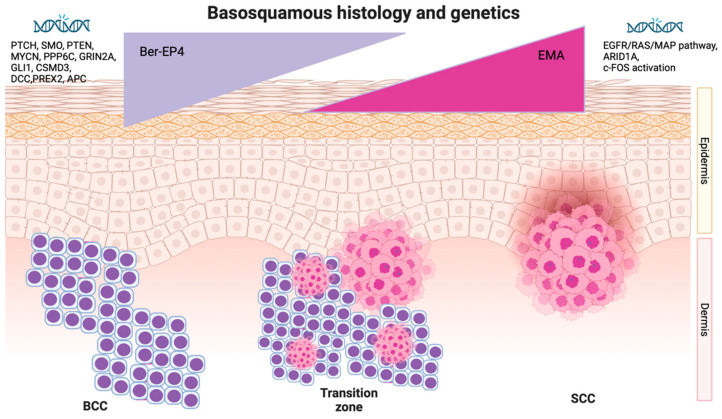
Basosquamous histological, immunohistochemical, and genetic features.

Numerous authors have proposed that the transition from basal to squamous cell carcinoma (BST) originates from epigenetic modifications [48]. The etiopathogenesis of BST remains a complex and debated matter, with potential involvement of various drivers and inducers such as SMO inhibitors (SMOi), activation of transcriptional inducers like c-FOS, and subsequent mutations such as ARID1A. Preclinical studies indicate that inhibiting SMO can prompt the shift of basal cell carcinoma to squamous cell carcinoma by promoting c-FOS activation of the EGFR/RAS/MAPK pathway. Conversely, inhibiting EGFR can reverse the effects of this transition [47,48]. More extensive research efforts are still required to fully understand this phenomenon. Figure 1 shows basosquamous histological, immunohistochemical, and genetic features. 

### 3.6. Biologic Behavior and Prognosis

In contrast to BCC, BSC exhibits a more aggressive biologic behavior that is nearly comparable to SCCs’ [2,5,18,30,41]. This “aggressiveness” is characterized by a more dynamic local tumor growth as well as a higher risk of recurrences and metastasis. As a consequence, these characteristics are associated with a worse prognosis as compared to the classic BCC [2,5].

According to Volkestein et al., the local recurrence rate of BSC—after wide surgical excision—reaches 45%, which is almost double that of BCC and SCC [18]. In a different study where Mohs’ micrographic surgery was performed, the topical recurrence rate for BSC dropped to 4–9% but remained higher than BCC (0.64%) and SCC (1.2%) [2,14,42,43,49]. 

The metastatic potential of BSC ranges between 4 and 8.4%, which is closer to that of SCC [2,42,50,51]. Ciazynska et al. [52] reported that 40% of patients diagnosed with BSC had a second skin neoplasm. This percentage is significantly higher than the corresponding 23% reported with other nonmelanoma skin cancers (NMSCs) and multiple lesions [7]. In this context, they concluded that BSC patients are more prone to the development of new primary skin cancers, and for this reason, they should be closely monitored [7].

### 3.7. Treatment of BSC

There are currently no standardized approved therapeutic guidelines for the management of BSCs. The rarity of these tumors along with the absence of robust literature data are the most reasonable explanations for the present scenario. Nevertheless, a variety of treatments have been used, with different degrees of efficacy. Superficial methods such as curettage and electrodesiccation have been used in the past but are not considered first line treatment due to their high recurrence rate. A multidisciplinary team (MDT) evaluation is always recommended.

#### 3.7.1. Wide Surgical Excision

Similarly to BCC and SCC, the first-line treatment for BCS is surgical excision, but surgical margins should be wider than those for low-risk BCC due to the infiltrative growth pattern of this tumor [53,54,55,56,57,58,59,60]. Favorable aesthetic outcome requires precise planning of the reconstructive technique [59]. Although a very high recurrence incidence of up to 45% has been reported after wide surgical excision, several studies [2,5,12,16,17,18,20,21,29,53] still recommend this approach as the first-line therapeutic option. Additionally, it is recommended that surgical excision be followed by the evaluation of lymph nodes and distant metastases, as well as, of course, close clinical followup for recurrence and metastasis [2,6,17].

#### 3.7.2. Mohs’ Micrographic Surgery (MMS)

According to recent studies, MMS is the best surgical option for BSCs, since it is associated with a lower rate of recurrence than wide surgical excision [17,29,30,54,55]. Analytically, Skaria et al. demonstrated an 8.9% recurrence rate with MMS, which is significantly higher when compared to the recurrence rates reported for BCCs and SCCs but significantly lower than the 45% observed with large surgical excision [54]. Even lower recurrence rates (4.9%) were reported by Allen et al. [55]. However, in order to formally endorse MMS as the preferred first-line treatment for BSCs, according to Oldbury et al., a number of logistical and budgetary obstacles must be resolved: (a) the inability to pre-operatively choose the ideal MMS candidates (i.e., clinical diagnosis of BSC instead of BCC) in everyday clinical practice; (b) higher costs of MMS compared to surgical excision; (c) more time-consuming process; and (d) its non-applicability in many centers worldwide [12].

#### 3.7.3. Sentinel Lymph Node Biopsy (SLNB)

The use of Sentinel Lymph Node Biopsy (SLNB) in the management of BSC has been recommended by some authors. However, there is ongoing controversy regarding whether SLNB should be routinely offered for BSCs to enable early diagnosis of occult nodal metastasis, staging, and treatment of subclinical node disease [17,32]. Kakagia et al. have suggested that a tumor size greater than 2 cm, along with lymphatic and perineural invasion, are significant factors in determining SLN (Sentinel Lymph Node) micrometastasis [56]. Yoshida et al. [57] propose that SLNB might be considered for specific high-risk groups, such as patients with a BSC larger than 3 cm located on the trunk or extremities. Given that the metastatic rate of BSC is still lower than that of malignant melanoma or Merkel cell carcinoma, routine use of SLNB for BSC is not generally recommended. However, further prospective controlled studies with a longer followup are required to validate any potential benefit of early SLNB for nodal spread and distant metastases [57].

#### 3.7.4. Radiotherapy

Several authors have proposed adjuvant radiotherapy (RT) for BSC in specific scenarios, such as when positive surgical margins prevent re-excision of the tumor or in cases with local lymph node metastasis [2,12,17,58]. There is a suggestion that radiotherapy, either as a standalone treatment or in combination with surgery, could be a suitable option for managing BSC when standard surgical excision or Mohs micrographic surgery (MMS) is not feasible [12,59,60]. This approach may be considered in situations where alternative surgical options are limited, aiming to enhance local control and manage potential residual disease. The American Society of Radiation Oncology recommend consideration of post-operative RT for gross perineural spread that is clinically or radiologically apparent. The use of definitive RT is discouraged for the treatment of SCC or BCC in patients with genetic conditions predisposing them to intensified radiosensitivity, such as ataxia telangiectasia, nevoid basal cell carcinoma syndrome (Gorlin syndrome), or Li–Fraumeni syndrome. Poorly controlled connective tissue disorders are a relative contraindication to treatment. For patients with BCC undergoing adjuvant RT, a dose of 6000 cGy (conventional fractionation [180–200 cGy/fx]) is recommended; for definitive treatment, 7000 cGy (conventional fractionation [180–200 cGy/fx]) is the standard [60].

#### 3.7.5. Antitumoral Drugs: Chemotherapy

BSC not eligible for locoregional therapies might be evaluated by the MDT, and a strategy including target therapy, immunotherapy, and, in rare cases, chemotherapy might be suggested. The patient journey, comorbidities, age, primary and node sites, symptoms, and the prevalence of either BCC or SCC behavior must be considered. In a few cases of locally advanced and metastatic BSCs, palliative chemotherapy has been used (adriamycine, cisplatin, carboplatin, paclitaxel) [61,62,63]. Kanzaky et al. [61] presented a case report of metastatic BSC treated with adriamycine and cisplatin. Sheen et al. described the complete regression of an extensive and locally destructive BSC involving the nasal septum and sinuses in a patient after a 4-month regimen of intra-arterial (IA) infusion of low-dose cisplatin [61]. Papageorgiou et al. presented a case of a metastatic BSC that was resistant to vismodegib treatment but showed complete remission following 3 months of carboplatin and paclitaxel [62].

#### 3.7.6. Sonic Hedgehog Inhibitors (HHIs)

Hedgehog inhibitors have revolutionized the treatment of BCC with impressive outcomes. However, few data are available for BSC, and there is controversy in the MDT about whether BSC should be treated as SCC with first-line immunotherapy or as BCC with first-line HHIs. Vismodegib and sonidegib are HHIs approved for the treatment of adults with metastatic BCC or locally advanced BCC that has recurred after surgery or adults who are not candidates for surgery or radiation. According to the available literature data, the risk of developing SCC in individuals treated with vismodegib is controversial [64,65,66,67,68]. Considering that BSC pathologically exhibits features of both BCC and SCC, treatment with vismodegib might bear the risk of progression of the SCC component of the tumor. Indeed, reports have been published on the development of SCC in patients treated with vismodegib, and a case-control study of 180 patients found an increased risk of SCC in patients treated with vismodegib [64]. On the other hand, the largest published cohort study of 1675 patients suggests that vismodegib is not associated with an increased risk of developing SCC, while a systematic and a narrative review of the literature concluded that the available evidence does not justify an association between vismodegib and the development of SCC [68,69]. In addition, there are very recent case reports involving a total of five patients that provide preliminary evidence that vismodegib may be effective in difficult-to-treat BSC (Table 1) [69,70,71]. In all cases, the lesions did not “transform” into pure squamous cell tumors but, on the contrary, regressed completely, and in two cases remission was maintained over a very long period [69,70,71]. Despite these positive data, in one case of basosquamous carcinoma treated with vismodegib, the drug had to be discontinued due to adverse effects before its efficacy could be evaluated [72]. In this sense, a post hoc analysis described the time to onset and severity of treatment emergent adverse events (TEAEs) in patients treated with the two HHIs. The results suggest a delayed onset of many common TEAEs and a lower incidence of muscle cramps, alopecia, and dysgeusia in patients treated with sonidegib 200 mg compared to vismodegib 150 mg [73].

Sonidegib is approved for the treatment of adult patients with recurrent locally advanced BCC who are not candidates for surgery or radiotherapy. The pivotal BOLT study (NCT01327053) [74,75] is an international, randomised, and double-blind phase 2 study that evaluated the long-term efficacy of sonidegib 200 and 800 mg once daily in all histological subtypes of BCC after 42 months. The primary endpoint was the objective response rate (ORR) [74,75]. A total of 230 patients were included; 36 (15.7%) with metastatic BCC and 194 (84.3%) with locally advanced BCC (aggressive, 112 of 194 [57.7%]; nonaggressive, 82 of 194 [42.3%]). The ORR at 42 months for patients with aggressive and nonaggressive locally advanced BCC was 59.5% (22 of 37) and 51.7% (15 of 29) for 200 mg [74,75], respectively. Approximately 50% of patients with nonaggressive subtypes in each group achieved an objective response [75]. The median time to response (TTR) for patients with an infiltrative subtype was 4.7 months (95% confidence interval, 1.9–6.6 months) for sonidegib 200 mg [75]. Recent studies [76,77] demonstrate the efficacy and safety of sonidegib in the treatment of BSC. The aggressive biological behavior and clinical course distinguish basosquamous carcinoma from other forms of BCC. In view of the greater risk of postoperative recurrence, some authors have recently investigated the neoadjuvant approach. Prof E. Dika [77] reported a clinical case of BSC on the scalp and zygomatic region treated with sonidegib at a dose of 200 mg daily in a neoadjuvant setting. After 8 months, the patient showed complete response of all lesions, and the MTB decided to proceed with excisional biopsy of the remaining zygomatic lesion.

Although the effect of HHIs on BSC treatment requires further investigation in larger controlled trials, HHIs such as vismodegib and sonidegib may represent a safe and effective first line treatment [78].

**Table 1:** Case reports (CR) presenting locally advanced (laBSC) and metastatic BSC (mBSC) treated with vismodegib and sonidegib.

Study	N° of CR	Skin Neoplasm	Treatment	Duration of Treatment (Months/Cycles)	Time to Complete Response (Months)	Durability of Response	Progression
McGrane et al. *Clin. Exp. Dermatol.* 2017 [68]	1	mBSC	Vismodegib 150 mg day	28 months	3	Complete response on primary BSC, partial response on metastasis	No
Sahuquillo-Torralba et al. *Indian J. Dermatol. Venereol. Leprol.* 2019 [69]	1	laBSC	Vismodegib 150 mg day	7 months	7	9 months after discontinuation of therapy	No
Apalla et al. *Eur Dermatol. 2019* [70]	2	laBSC	Vismodegib 150 mg day	6 months	6	12 and 18 months after discontinuation of therapy	No
Pirruccello et al. *BMJ Case Rep.* 2023 [71]	1	laBSC	Vismodegib 150 mg day + Cemiplimab 350 mg every 3 weeks	31 cycles of Cemiplimab	21	/	No
Toffoli et al. *Dermatol. Ther.* 2022. Jun, 35(6), e15436. [76]	2	laBSC	Sonidegib 200 mg day	On course	6	Therapy will be continued until disease progression or unacceptable toxicity	No
Dika et al. *Exp Dermatol.* 2023. Jul 11. [77]	1	laBSC	Sonidegib 200 mg day + surgery	8 months of Sonidegib	8	Complete remission after 6 months	No

#### 3.7.7. Checkpoint Inhibitors

Immune checkpoint inhibitors, specifically anti-CTLA4, anti-PD1, and anti-PDL-1 antibodies, are commonly used to treat skin cancers, including melanoma, SCC, BCC, and Merkel cell carcinoma. These tumors and BCC have similarly high rates of malignant mutations and T cell infiltration. In cases of locally advanced, recurrent, or metastatic SCC where options for locoregional treatment are limited, the recommended therapeutic approach is the use of anti-PD1 cemiplimab, especially in patients who have exhausted other treatment options. For locally advanced BCC (laBCC), cemiplimab serves as a second-line treatment when curative surgery or radiation is not viable. This is applicable to patients who have experienced progression on hedgehog pathway inhibitors (HHIs), are intolerant to HHI therapy, or have not objectively responded after months of HHI therapy. Studies on cemiplimab have demonstrated a favorable effectiveness and tolerability profile, albeit with a shorter average length of response compared to SCC [71,79,80,81]. In a Phase II study led by Stratigos in 2021, which involved 84 patients experiencing progression on hedgehog inhibitors (HHI), 31% of patients achieved an objective response rate, with 6% of those responses being complete [81]. Subsequent studies confirmed the positive effectiveness and tolerability profile of cemiplimab, although the duration of response in BCC was observed to be shorter than in SCC. In a real-world retrospective analysis of cemiplimab in locally advanced SCC, among 25 patients, 52% demonstrated an objective response (3 complete and 10 partial responses), with 76% achieving disease control [82]. In a multicenter retrospective study by GK et al., involving 29 patients with locally advanced or metastatic BCC, an overall response rate (ORR) of 31% was observed in metastatic BCC and 69% in locally advanced BCC [83]. Cemiplimab also offers an alternative for patients with BSC resistant or intolerant to hedgehog inhibitors (HHI). Given the intricate pathobiology of BSC, there is interest in targeting both components, involving tumor immune dysregulation and abnormal activation of hedgehog signaling. Patel et al. reported the benefit of cemiplimab ± HHIs in 16 patients [84]. Colombo et al. documented two cases of advanced synchronous BCC/cSCC of the head and neck treated with combined therapy using cemiplimab and sonidegib, achieving significant clinical benefit and long-term responses without major adverse events [85]. Other treatments options such as the PD-L1 inhibitors pembrolizumab and nivolumab have been used for the treatment of advanced-metastatic BCCs and SCCs, but there are few literature data for their use in advanced BSC [72,73,81]. Table 2 shows three case reports presenting locally advanced and metastatic BSC treated with checkpoint inhibitors. 

## 4. Conclusions

Basaloid squamous cell carcinoma (BSC), a contentious entity within nonmelanoma skin cancers, shares features with both basal cell carcinoma (BCC) and squamous cell carcinoma (SCC) at clinical, dermoscopical, confocal, and histological evaluation. Clinical examination alone cannot distinguish BSC from BCC, but dermoscopy and LC-OCT offer potential for more accurate diagnosis by revealing characteristics invisible to the naked eye. Definitive diagnosis relies on histology, often aided by deep incisional biopsies and immunohistochemistry, particularly Ber-EP4 staining. The absence of standardized treatment protocols for BSC necessitates prospective studies comparing various options to establish a consensus on ideal management. Surgical approaches, notably wide excision and Mohs’ micrographic surgery, are favored by many clinicians. Controversies persist around the use of sentinel lymph node biopsy (SLNB), radiation therapy, and imaging monitoring, especially in cases with suspicious features like a tumor size exceeding 2 cm or evidence of perineural and lymphatic invasion. Radiotherapy could have a supportive role, postoperatively, when re-excision is not possible or not allowed by the patient. Finally, new treatment prospective with sonic hedgehog inhibitors and checkpoint inhibitors must be thoroughly investigated, with large controlled trials, since it may offer an alternative solution to irresectable or difficult-to-treat locally advanced cases of BSC.

## Figures and Tables

**Table 2:** Case reports (CR) presenting locally advanced (laBSC) and metastatic BSC (mBSC) treated with checkpoint inhibitors.

Study	N° of CR	Skin Neoplasm	Treatment	Duration of Treatment (Cycles)	Time to Complete Response (Cycles)	Durability of Response	Progression
Pirruccello et al. *BMJ Case Rep.* 2023 [71]	1	laBSC	Vismodegib 150 mg day + Cemiplimab 350 mg every 3 weeks	31 cycles	21 cycles	/	No
Borradori et al. *Br. J. Dermatol.* 2016 [72]	1	laBSC resistant to Vismodegib	Nivolumab 3 mg/kg, every 2 weeks	4 cycles	4 cycles	/	No
Gambichler et al. *J. Eur. Acad. Dermatol. Venereol.* 2022 [80]	1	laBSC resistant to Sonidegib	Cemiplimab 350 mg every 3 weeks	/	4 cycles	/	No

## Data Availability

No new data were created or analyzed in this study. Data sharing is not applicable to this article.
